# Supplementary material for: Associations between serum retinol and all-cause mortality among adults with prediabetes and diabetes: A cohort study
Source: PLoS One. 2024 Feb 2;19(2):e0297552. doi: 10.1371/journal.pone.0297552 (PMC10836695; doi:10.1371/journal.pone.0297552)
Supplement: S1 File — (DOCX) [file pone.0297552.s003.docx]

**Supplementary File**

31,509 participants from NHANES 2001-2006

1183 participants pregnant or lactating

20,494 Participants aged <40 years

5342 Participants without prediabetes or diabetes

4490 participants aged ≥40 years with prediabetes or diabetes

254 Participants without serum retinol

2582 participants with prediabetes

1654 participants with diabetes

**Supplementary Figure 1. Flow chart of the study population**


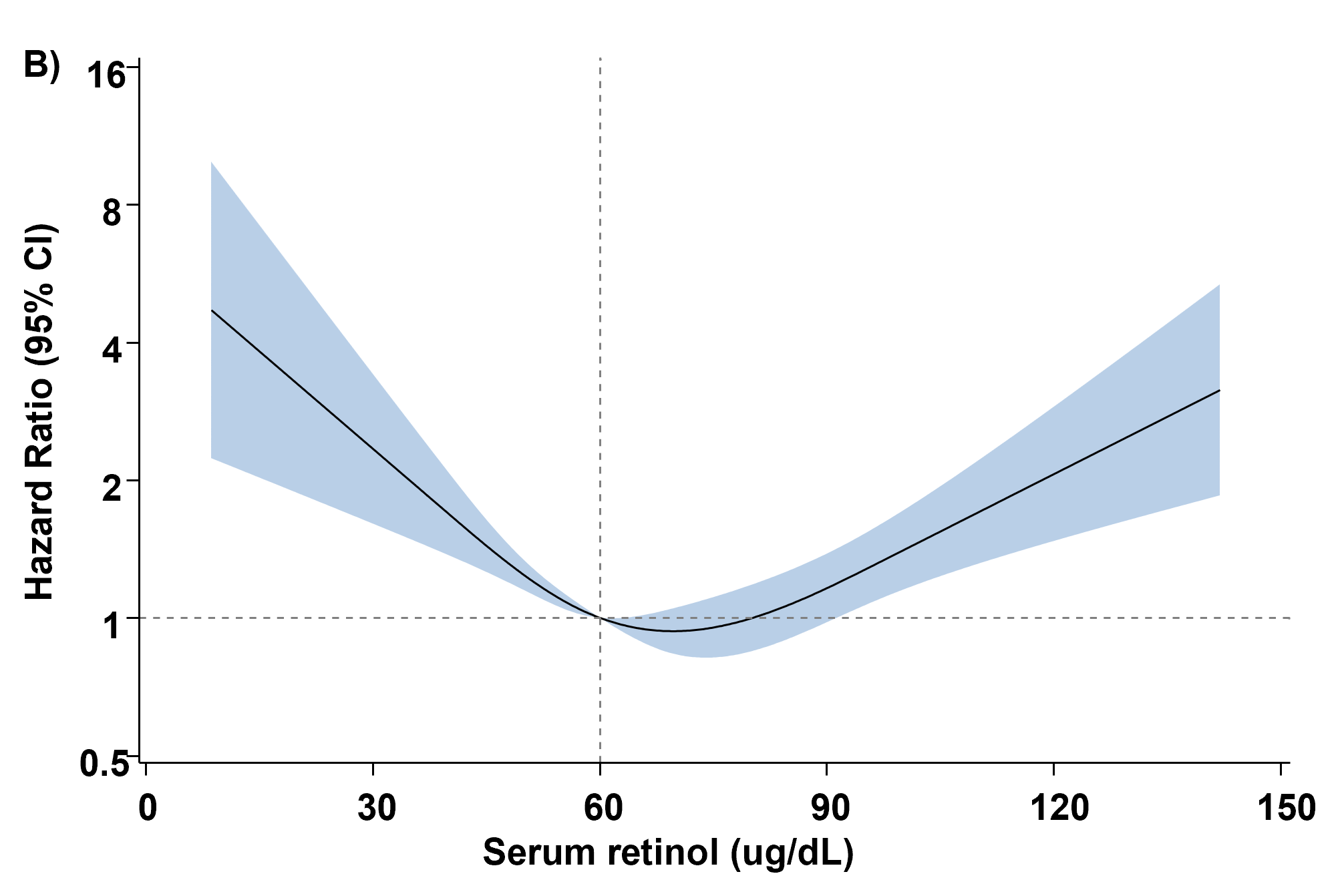

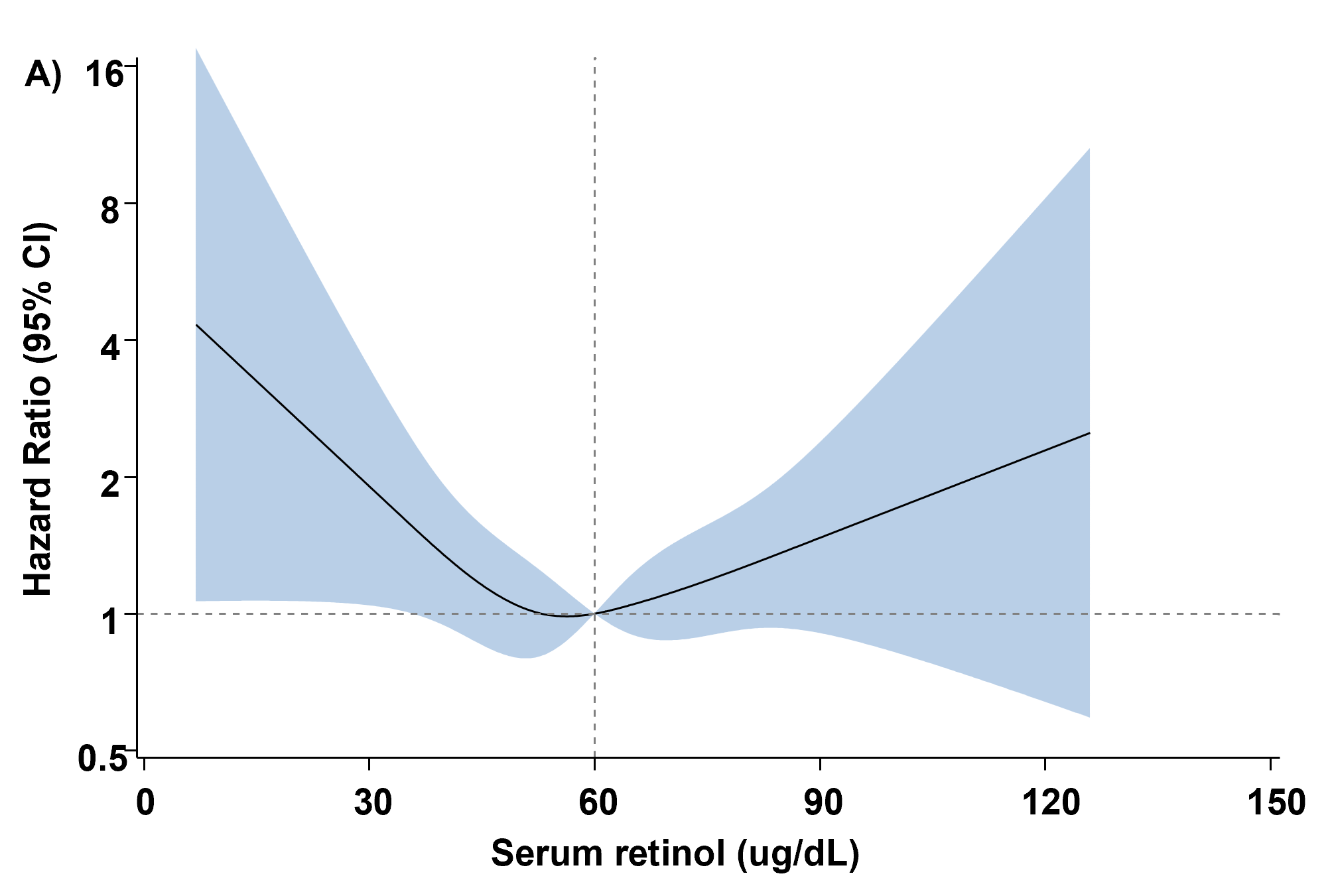

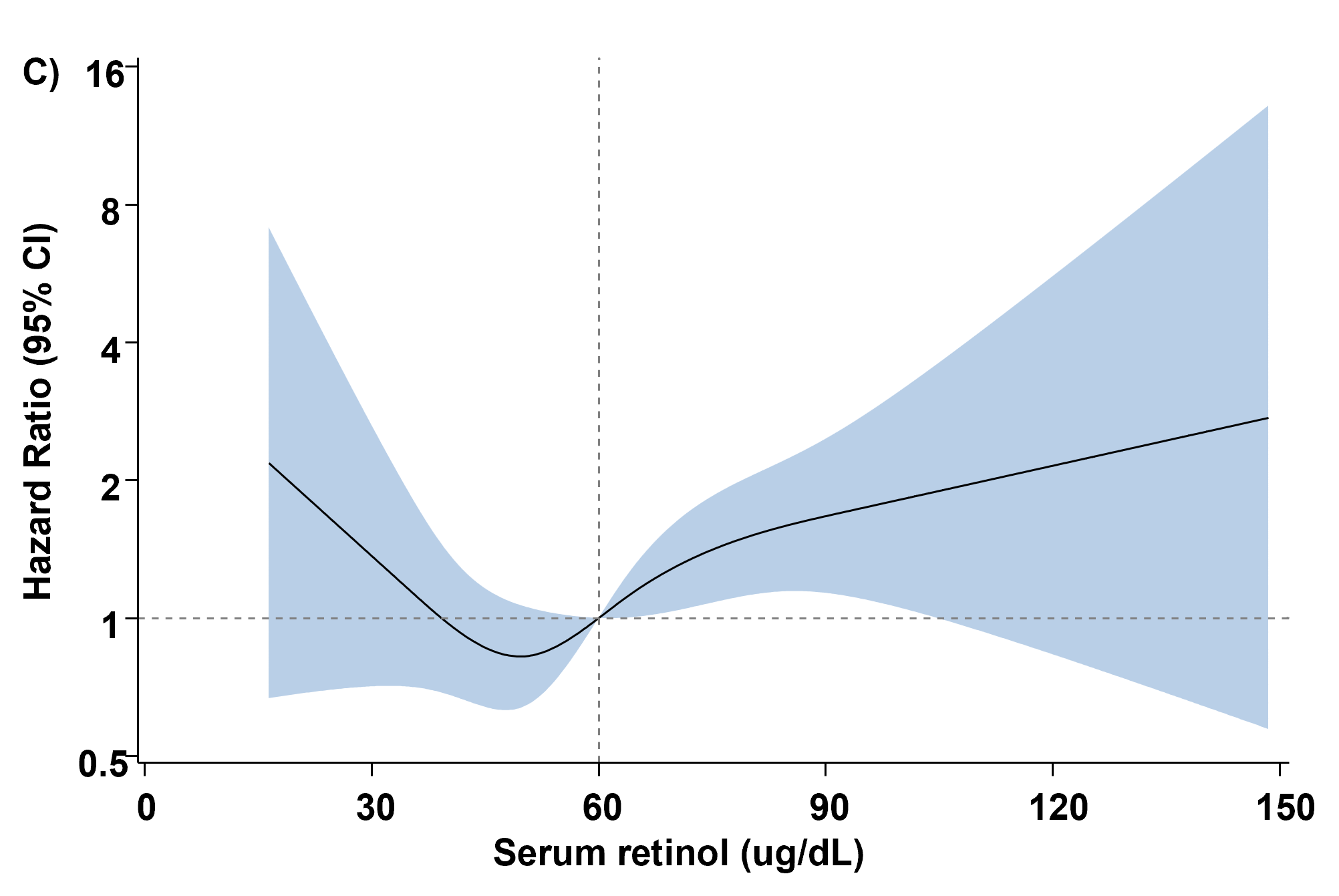

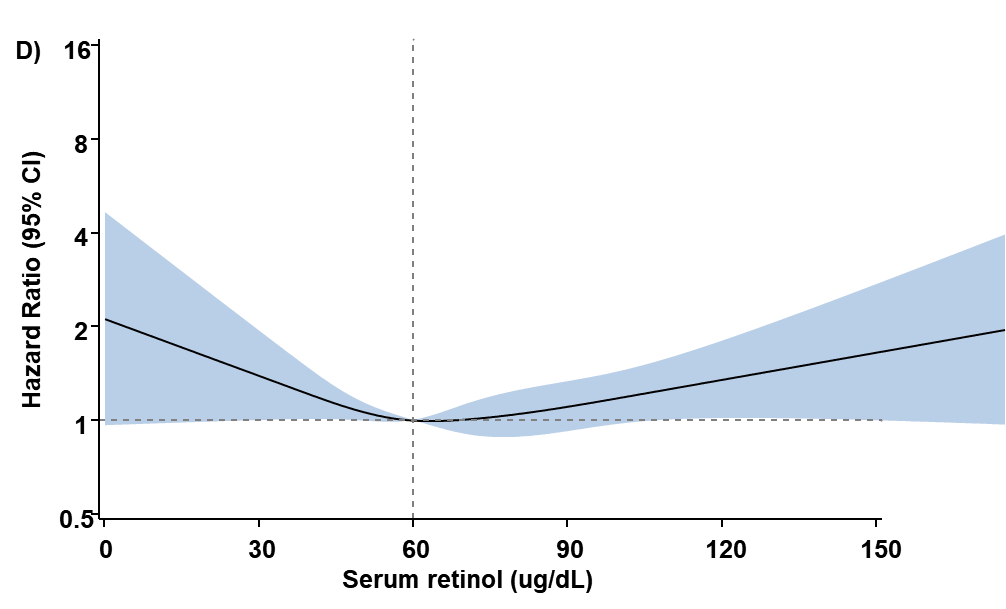


**Supplementary Figure 2. The association between serum retinol (μg/dL) and all-cause mortality among participants aged 40-59 years with prediabetes (A), aged ≥60 years with prediabetes (B), aged 40-59 years with diabetes (C), and aged ≥60 years with diabetes (D).**

Adjusted for age, sex, ethnicity, education, smoking status, alcohol consumption, physical activity, body mass index, hypertension, total cholesterol. Among participants aged 40-59 years with prediabetes (A), *P*-value for non-linear association was 0.078 and *P*-value for linear association was 0.952. Among participants aged ≥60 years with prediabetes (B), *P*-value for non-linear association was <0.001 and *P*-value for linear association was 0.600. Among participants aged 40-59 years with diabetes (C), *P*-value for non-linear association was 0.134 and *P*-value for linear association was 0.064. Among participants aged ≥60 years with diabetes (D), *P*-value for non-linear association was 0.042 and *P*-value for linear association was 0.570.

**Supplementary Table 1. Hazard ratios (HR) and 95% confidence intervals (CI) for all-cause mortality by serum retinol among prediabetes and diabetes separately.**

| **Serum retinol** | **No. of participants** | **Person years** | **No. of events** | **HR (95% CI)** |
| --- | --- | --- | --- | --- |
| **Among participants with prediabetes** | | |  |  |
| <50 μg/dL | 592 | 7600 | 215 | 1.37 (1.09-1.73) |
| 50-60 μg/dL | 633 | 8425 | 224 | Reference |
| 60-70 μg/dL | 584 | 7604 | 227 | 0.98 (0.79-1.21) |
| 70-80 μg/dL | 391 | 5282 | 142 | 0.84 (0.65-1.10) |
| 80+ μg/dL | 382 | 4612 | 185 | 1.23 (0.98-1.54) |
| **Among participants with diabetes** | | |  |  |
| <50 μg/dL | 401 | 4879 | 176 | 1.28 (0.98-1.69) |
| 50-60 μg/dL | 370 | 4618 | 178 | Reference |
| 60-70 μg/dL | 336 | 3814 | 183 | 1.10 (0.81-1.48) |
| 70-80 μg/dL | 238 | 2787 | 134 | 0.98 (0.71-1.37) |
| 80+ μg/dL | 309 | 3248 | 203 | 1.21 (0.90-1.65) |

Adjusted for age, sex, ethnicity, education, smoking status, alcohol consumption, physical activity, waist circumference, hypertension, total cholesterol.

**Supplementary Table 2. Hazard ratios (HR) and 95% confidence intervals (CI) for cardiovascular and non-cardiovascular disease mortality by serum retinol among prediabetes and diabetes separately.**

| **Serum retinol** | **No. of participants** | **Person years** | **No. of events** | **HR (95% CI)** |
| --- | --- | --- | --- | --- |
| **For cardiovascular disease death** | | |  |  |
| **Among participants with prediabetes** | | |  |  |
| <50 μg/dL | 592 | 7600 | 67 | 1.28 (0.84-1.97) |
| 50-60 μg/dL | 633 | 8425 | 84 | Reference |
| 60-70 μg/dL | 584 | 7604 | 75 | 0.83 (0.53-1.30) |
| 70-80 μg/dL | 391 | 5282 | 46 | 0.78 (0.49-1.26) |
| 80+ μg/dL | 382 | 4612 | 67 | 1.25 (0.84-1.86) |
| **Among participants with diabetes** | | |  |  |
| <50 μg/dL | 401 | 4879 | 63 | 1.00 (0.63-1.58) |
| 50-60 μg/dL | 370 | 4618 | 82 | Reference |
| 60-70 μg/dL | 336 | 3814 | 91 | 1.16 (0.72-1.86) |
| 70-80 μg/dL | 238 | 2787 | 53 | 0.72 (0.43-1.19) |
| 80+ μg/dL | 309 | 3248 | 103 | 1.44 (0.99-2.10) |
| **For non-cardiovascular disease death** | | |  |  |
| **Among participants with prediabetes** | | |  |  |
| <50 μg/dL | 592 | 7600 | 148 | 1.16 (0.91-1.48) |
| 50-60 μg/dL | 633 | 8425 | 140 | Reference |
| 60-70 μg/dL | 584 | 7604 | 152 | 1.15 (0.87-1.51) |
| 70-80 μg/dL | 391 | 5282 | 96 | 1.03 (0.73-1.46) |
| 80+ μg/dL | 382 | 4612 | 118 | 1.24 (0.89-1.73) |
| **Among participants with diabetes** | | |  |  |
| <50 μg/dL | 401 | 4879 | 113 | 1.62 (1.11-2.36) |
| 50-60 μg/dL | 370 | 4618 | 96 | Reference |
| 60-70 μg/dL | 336 | 3814 | 92 | 1.03 (0.66-1.59) |
| 70-80 μg/dL | 238 | 2787 | 81 | 1.20 (0.79-1.82) |
| 80+ μg/dL | 309 | 3248 | 100 | 0.97 (0.67-1.40) |

Adjusted for age, sex, ethnicity, education, smoking status, alcohol consumption, physical activity, body mass index, hypertension, total cholesterol.
